# Instantaneous and non-destructive relative water content estimation from deep learning applied to resonant ultrasonic spectra of plant leaves

**DOI:** 10.1186/s13007-019-0511-z

**Published:** 2019-11-07

**Authors:** María Dolores Fariñas, Daniel Jimenez-Carretero, Domingo Sancho-Knapik, José Javier Peguero-Pina, Eustaquio Gil-Pelegrín, Tomás Gómez Álvarez-Arenas

**Affiliations:** 1 Department of Food Technology, Universitat Politècnica de València (UPV), Valencia, Spain; 2 Cellomics Unit, Cell & Developmental Biology Area, Centro Nacional de Investigaciones Cardiovasculares (CNIC), Madrid, Spain; 3 Unidad de Recursos Naturales, Centro de Investigación y Tecnología Agroalimentaria Gobierno de Aragón (CITA), Zaragoza, Spain; 4 Sensors and Ultrasonic Technologies Department, Information and Physics Technologies Institute, Spanish National Research Council (CSIC), Madrid, Spain

**Keywords:** RWC, Ultrasounds, Machine learning, Plant leaves, Irrigation, NC-RUS

## Abstract

**Background:**

Non-contact resonant ultrasound spectroscopy (NC-RUS) has been proven as a reliable technique for the dynamic determination of leaf water status. It has been already tested in more than 50 plant species. In parallel, relative water content (RWC) is highly used in the ecophysiological field to describe the degree of water saturation in plant leaves. Obtaining RWC implies a cumbersome and destructive process that can introduce artefacts and cannot be determined instantaneously.

**Results:**

Here, we present a method for the estimation of RWC in plant leaves from non-contact resonant ultrasound spectroscopy (NC-RUS) data. This technique enables to collect transmission coefficient in a [0.15–1.6] MHz frequency range from plant leaves in a non-invasive, non-destructive and rapid way. Two different approaches for the proposed method are evaluated: convolutional neural networks (CNN) and random forest (RF). While CNN takes the entire ultrasonic spectra acquired from the leaves, RF only uses four relevant parameters resulted from the transmission coefficient data. Both methods were tested successfully in *Viburnum tinus* leaf samples with Pearson’s correlations between 0.92 and 0.84.

**Conclusions:**

This study showed that the combination of NC-RUS technique with deep learning algorithms is a robust tool for the instantaneous, accurate and non-destructive determination of RWC in plant leaves.

## Background

Most common methods to asses plant water status, through the measurement of either relative water content or water potential [1–4], are destructive techniques that preclude repetitive measurements in a given tissue [5]. Attempts to find a non-invasive technique suitable for the study of dynamic water changes within the same plant tissue have been a challenge during the last decades. In this sense, thermocouple psychrometers have been successfully used for measuring plant water potential [6]. However, their complexity installation might not be useful for a quick leaf monitoring [7–9]. Methods such as infrared thermometry [7] or canopy reflectance [8, 9] can also be used for plant water continuous estimations. Nevertheless, their accuracy is highly reduced by plant architecture [10], making these techniques more appropriate for crop science [7] than for accurate physiological measurements. Another set of techniques to estimate plant water status are based on the continuous monitoring of turgor pressure changes [11, 12]. In this sense, the ball tonometry method estimates the dynamic changes in plant water status by applying an external pressure on plant cells [12]. A requirement of this technique is that cell walls must be relatively thin, constituting a strong limitation for its widespread use. By contrast, the high-precision pressure probe developed by Zimmermann et al. [13] which allows the online monitoring of water relations in a great variety of species, requires a continuous contact with the leaf surface, not allowing the completely free transpiration of the leaf. Finally, the reflectivity in microwave L-band has been proven to estimate accurately the water content in poplar [14]. This technique takes advantage of the development of digital cordless telephony (DCT) but its use in leaves with different sizes implies the fabrication of different types of antennas.

**Fig. 1:**
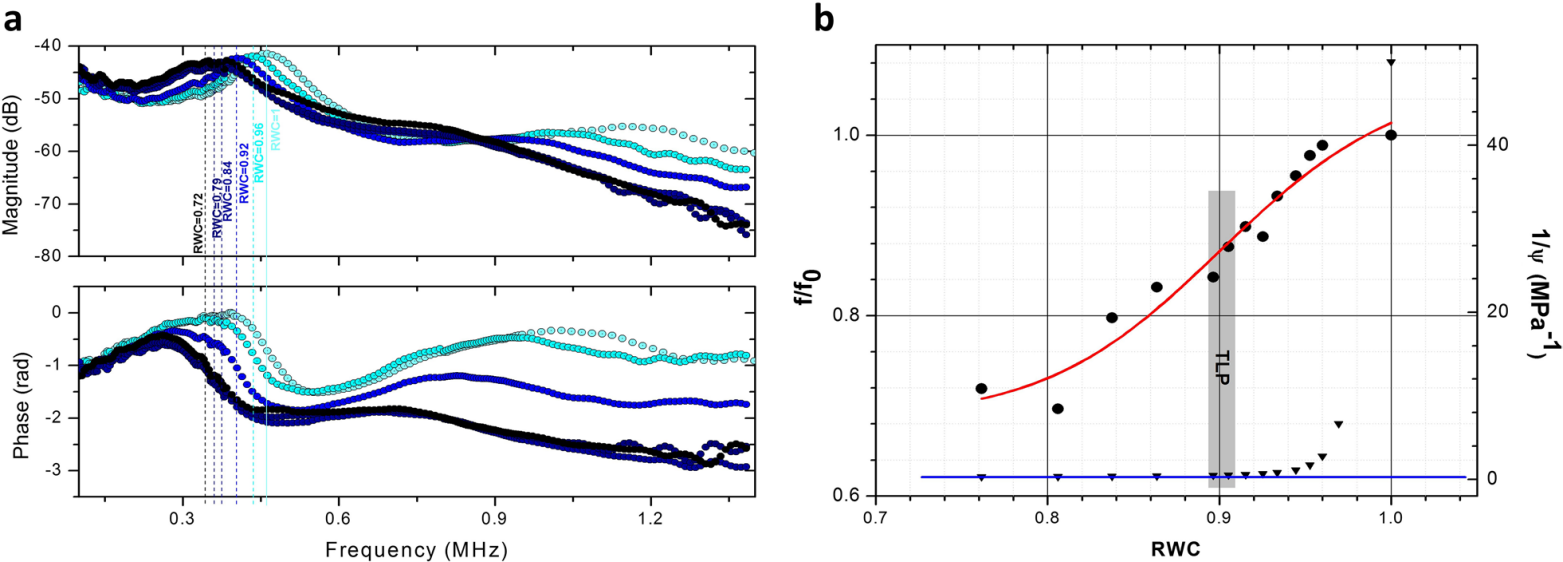
RWC measurements using NC-RUS. **a** Transmission coefficient spectra measured using NC-RUS technique in a detached *Viburnum tinus* leaf while drying at RWC values of 1, 0.96, 0.92, 0.84, 0.79 and 0.72. **b** Relationship between RWC values and f/f0 obtained through the NC-RUS technique (circles) and relationship between RWC values and the inverse of water potential (1/Ψ) obtained with the p–v curves (triangles) for *Viburnum tinus* leaf. The shaded rectangle marks the TLP on both relationships

Among all these plant-based methods, non-contact resonant ultrasound spectroscopy (NC-RUS) has been proven as a non-destructive, non-invasive and rapid method for the dynamic determination of leaf water status [15]. NC-RUS technique excites thickness resonances in plant leaves, using ultrasonic waves in the [0.15–1.6] MHz frequency range (Fig. 1a). These thickness resonances are sensitive to leaf microstructure, composition and water status in the leaf [16]. Later work was developed in order to compare these ultrasonic measurements to well-established techniques such as pressure–volume curves in drying experiments on several species [17]. As a result, it was observed that relative water content values at turgor loss point (RWC_TLP_) obtained using the NC-RUS technique did not show any significant difference compared to those obtained using p–v curves approximation (Fig. 1b). In parallel, an effective medium approach was used to interpret the transmission coefficient spectra of the leaf and not only the resonant frequency value [18, 19]. This model allows an accurate determination of effective properties of the leaf such as thickness, volumetric density, velocity of ultrasound through the leaf, ultrasound attenuation coefficient, acoustic impedance (velocity and density product), among others. These estimations can be improved by using a layered leaf model, closer to the real leaf structure considering the leaf formed by two acoustically different layers [20, 21]. Some results from these studies showed that the transmission coefficient of the ultrasonic waves is sensitive not only to changes in different abiotic stimuli studied, but also on the specific properties of the leaves of particular species, the environmental conditions under which the plant was grown and the actual state of the leaf and consequently, of the plant itself [22, 23].

All these physical parameters obtained from ultrasonic measurements can provide critical information about the leaf properties and their relation with the leaf water status, specifically with the relative water content (RWC) [24]. RWC is an appropriate measure of plant hydration state and generally accepted as a proxy of the physiological consequence of cellular water deficit. However, estimation of RWC is still bound to traditional, destructive and time-consuming techniques relying on mass differences of the same leaf sample at different hydration states (including turgid and dry states as references) [25]. Despite the relative simplicity of classical methods, RWC measurements require from careful work in a controlled environment that is usually far away from field-grown locations. The need of sample transportations, together with the ephemeral character of fresh leaves; hence imply important limitations to achieve a successful and reliable estimation of RWC. In fact, important indicators such as RWC_TLP_ requires of a whole set of RWC values for its computation.

This work represents a step forward in the instantaneous estimation of RWC in a non-invasive, non-destructive and rapid way. The proposed approach uses NC-RUS measurements and apply advanced machine learning regression and especially deep-learning method to infer RWC value from one single measurement. Deep convolutional neural networks (CNN) [26] is the most recent major advance in machine learning and computer vision. CNNs can automate the critical steps of feature extraction and selection by learning high-level features based on spatial relationships in data, and thus seemed well suited to exploit the spectral nature of NC-RUS data for the analysis of water status in plants. Although deep learning has proven to be a very effective tool for detection, segmentation, classification, and regression problems, its application in plant science is still limited. A small amount of works have shown the advantages of these techniques for image-based plant phenotyping tasks [27–29], but to the best of our knowledge, the only attempt to use machine learning to estimate plant water status was carried out in plant fields from multispectral imagery and using simple neural networks [30]. In this work, we use deep neural networks to estimate water content of plants from NC-RUS measurements on individual leaves from one single measurement.

## Results

Table 1 summarize obtained values of R and RMSE (used to measure the efficiency) from the different methods applied. Results display high correlations and small errors for both machine-learning approaches when using all augmented data: N = 1960. Results remain almost the same when evaluating the mean prediction of the seven different interpolated versions corresponding to the same leaf and hydration state: N = 280. In fact, there is no statistical differences between RWC predictions obtained with the different interpolated versions as shown in Additional file 1: Figure S1a, supporting the robustness of our approach and prediction models against noise and/or measurement imprecisions.

**Table 1:** Results of Pearson’s correlations (R) and root mean squared errors (RMSE) comparing predictions under the machine learning approaches proposed and the experimentally measured RWC values

Number of ultrasonic signals	Method	R	RMSE
1960	Random forest	0.8400	0.0591
1960	Convolutional neural network	0.9225	0.0407
280	Random forest	0.8453	0.0585
280	Convolutional neural network	0.9228	0.0406

Additional file 1: Figure S1b displays the comparison between RF and CNN results: the latter clearly excels in performance, confirmed by lower mean prediction errors obtained with the CNN approach. RF results suggest that the four NC-RUS-derived parameters contain essential information related to leaf water status, as previously reported in the literature. However, the use of the complete spectral data increases the exactness of RWC predictions. Therefore, NC-RUS information discarded so far when using only the established derived-parameters, although not core contains relevant information on leaf structure and corresponding water status. The use of CNNs allows not only to analyze the entire spectral information, but to do so by maintaining the continuous/sequential configuration of NC-RUS signals in the frequency range, rather than treating different variables as independent or unrelated.

**Fig. 2:**
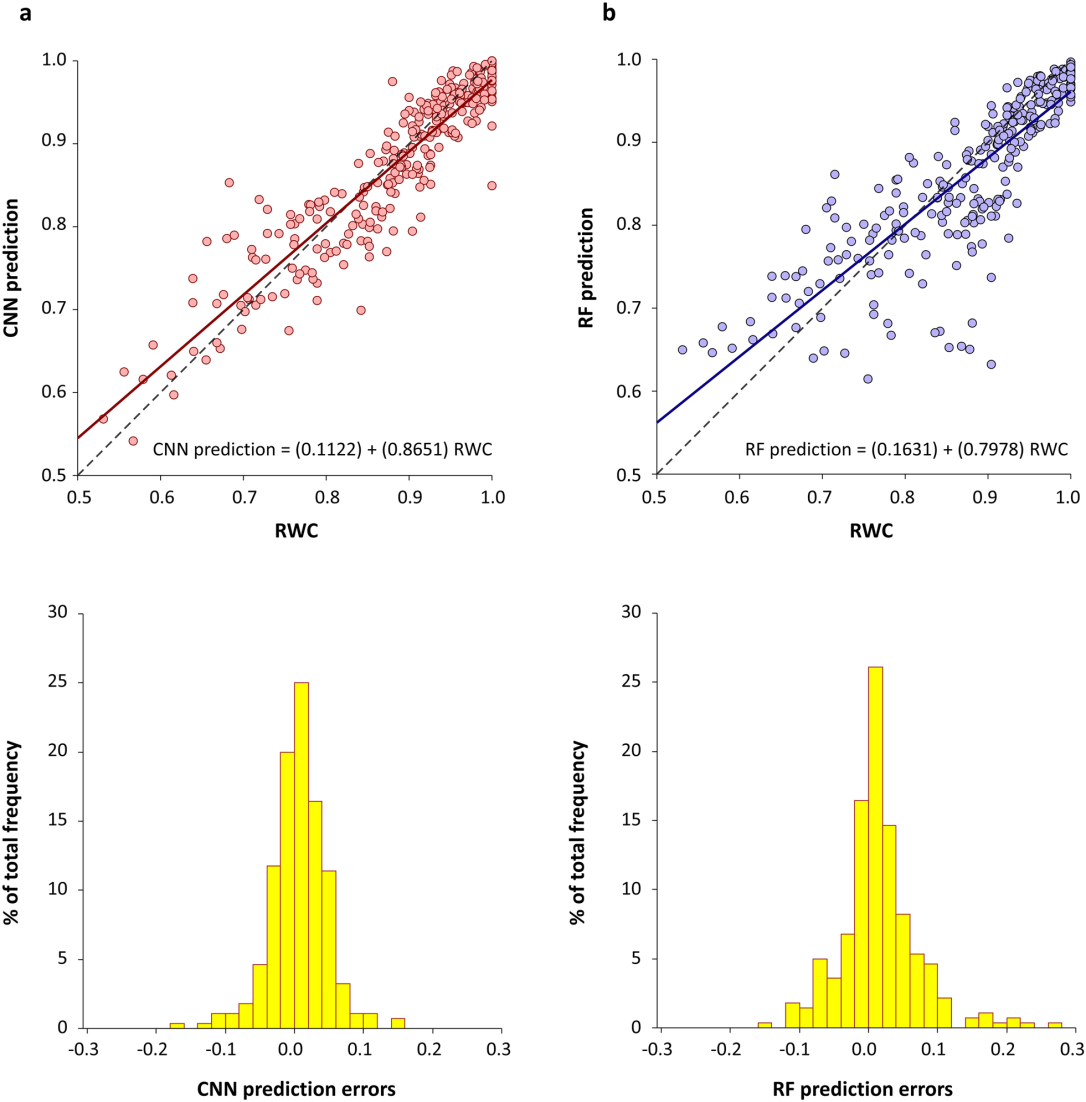
RWC estimation results. Comparison between RWC values and predictions performed with CNN (**a**) and RF (**b**) approaches. Dotplots (top) display actual RWC values and predictions, including linear regression lines (red/blue) and the reference line for a perfect regression (dashed black). Each dot corresponds to one interpolated version of NC-RUS data sample. Histograms (bottom) show the distribution of prediction errors

Figure 2 shows the obtained correlation between estimated and measured RWC values for CNN (Fig. 2a) and RF models (Fig. 2b). The linear regressions show a deviation from the perfect regression in both cases (CNN prediction = 0.8651·RWC + 0.1122; RF prediction = 0.7978·RWC + 0.1631), but as mentioned earlier, CNN performance is higher. Additionally, the distributions of relative prediction errors are centered around zero in both cases. It is worth mentioning that the lower number of measurements below RWC = 0.75, which correspond to the noisiest measurements, deviates the regression line from the optimal values. Indeed, the linear regression between the RWC [0.75–1] values is very close to the perfect regression line (CNN prediction = 0.9736·RWC + 0.0109; RF prediction = 1.0186·RWC + 0.0424).

## Discussion

The proposed technique for an instantaneous estimation of RWC through non-invasive and non-destructive ultrasonic measurements combined with machine learning approaches has been proved successfully in *Viburnum tinus* leaves.

In general, transmission coefficient spectra obtained using the NC-RUS technique are able to monitor changes in the water status of leaves connected or detached to the plant rapidly without direct contact. In this work, we combined this ultrasonic technique with two different machine-learning algorithms in order to translate that information directly into a well-known and widely used parameter as RWC. Our main aim is to obtain RWC instantaneously, avoiding the normalization process and hence the need of previously knowing the fresh and dried mass of the sample under study.

A total of 280 measurements from *V. tinus* leaves covering different hydration states were used in this work, comprised of NC-RUS transmission coefficient spectra and their corresponding RWC values measured experimentally. Sixty-three percent of the measurements corresponded to RWC values in the range of 1 to 0.88 (above turgor loss point, TLP), while the rest (37%) are distributed between 0.88 and 0.52 (below TLP). Although we had enough data points to achieve good correlation models throughout the whole RWC range (Fig. 2), we mainly focus the measurements above TLP as is the range for many plant physiological processes like plant growth or gas exchange.

**Fig. 3:**
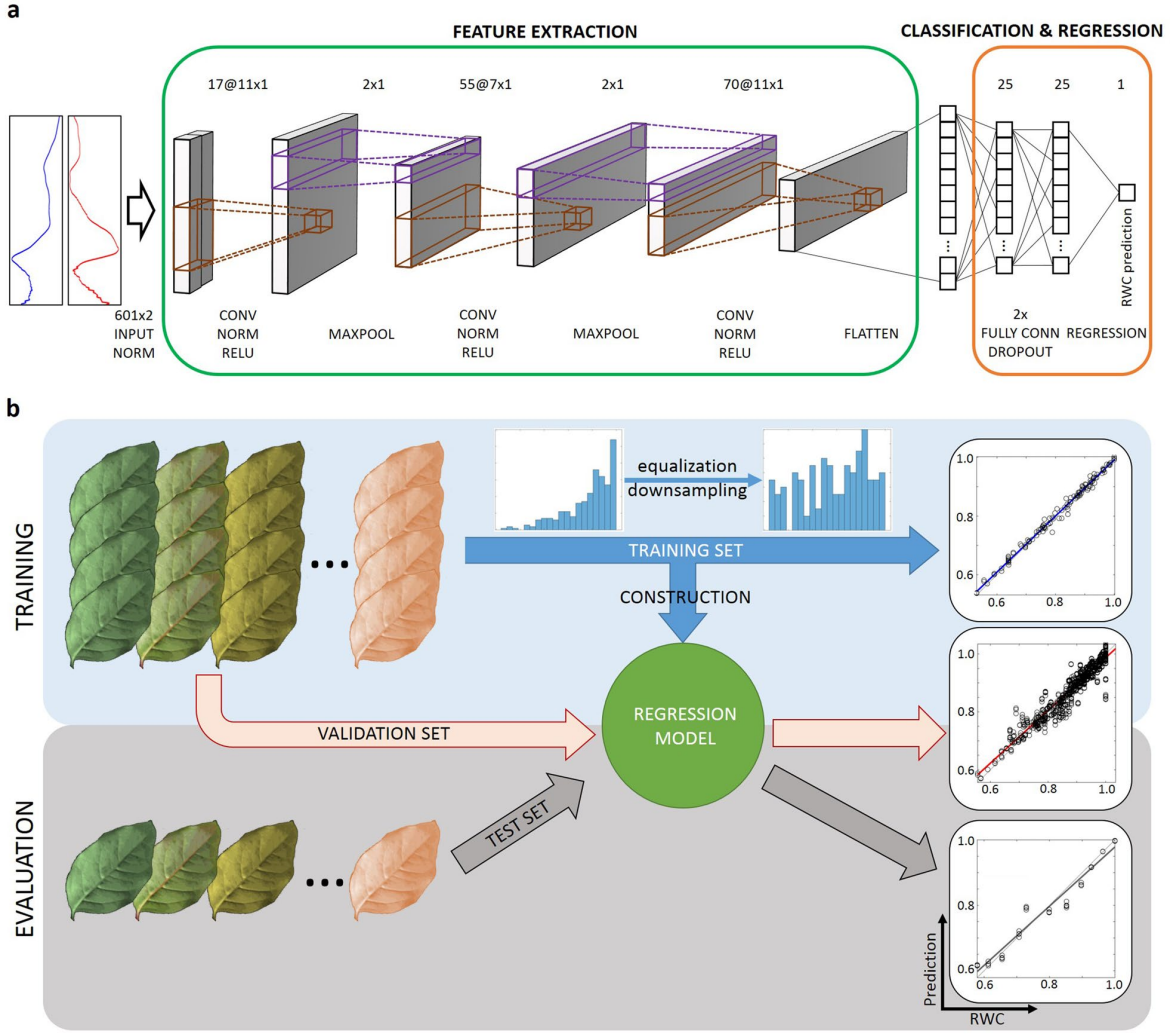
Deep learning architecture and evaluation strategy. **a** CNN architecture to predict RWC values from non-contact resonant ultrasound spectroscopy measurements (magnitude and phase). **b** Graphical representation of machine learning strategy to train and test the system (leafOO-CV)

We processed the magnitude and phase spectra using 1-dimensional CNNs (Fig. 3a) to estimate RWC values of leaves at each drying state. Additionally, four NC-RUS-derived parameters that probed to contain important structural information were also used in parallel to perform the prediction using random forest (RF) as machine learning method: maximum spectral magnitude and the corresponding frequency, phase and bandwidth. The generation and evaluation of regression models were carried out mimicking a representative practical scenario where a RWC-prediction model was constructed using measurements from a set of leaves with different hydration states. This set of leaves was afterwards used to estimate the RWC of completely new leaves, with unknown hydric state, that were never used for training that model (Fig. 3b). Therefore, training and test sets were created treating leaves separately.

Both approaches excelled in performance: results suggest that RWC can be determined immediately from transmission coefficient spectra measured directly over leaves by using CNN previously trained with data from plants on the same location. Moreover we assumed, based on our previous work, that four main parameters derived from coefficient spectra (maximum magnitude of the transmission coefficient, phase and frequency at which this maximum is located and the bandwidth at 6 dB) contained information enough about the water status of the leaf to train and estimate its RWC value. This assumption was successfully revealed on the RF results, whose correlation is slightly below the one performed using CNN. The main advantage of the RF-based approach is that the frequency range of the ultrasonic transmission coefficient needed is narrower and thus the measurement only require one pair of ultrasonic sensors.

## Conclusions

We proposed herein a new tool to estimate instantaneously RWC from ultrasonic measurements using NC-RUS technique in a non-destructive and non-invasive way applying two different machine-learning algorithms (CNN and RF) previously trained with experimental data coming from leaves within the same species and location. Although both algorithms excelled in performance, we consider that RF resulted more convenient since is able to predict RWC values using only one pair of ultrasonic transducers centered at the same working frequency. This might be translated into a faster, easier and cheaper application in the field.

Further work on collecting RWC-ultrasonic experimental data from different species or same species at different locations must be done in order to evaluate the suitability of applying transfer-learning methods, which can lead to a big improvement on the scalability of this technique.

Altogether, NC-RUS and the proposed RWC estimation method have the potential of becoming a rapid and robust tool to measure the hydration state of plants, which may provide a breakthrough in the irrigation scheduling of agriculture systems.

## Materials and methods

### Plant material

*Viburnum tinus* leaves were collected from The Royal Botanical Garden of Madrid (40° 24′ 40″ N, 3° 41′ 30″ W) steadily during 18 months. The easy availability of *V. tinus* leaves throughout the year and the high accuracy of NC-RUS measurements in this species, promoted its selection as plant material for this study. In the early morning, branches were collected, rapidly introduced in plastic containers with water in order to ensure a water–vapor saturated atmosphere and carried to the laboratory. Once in the lab, shoots were re-cut under water to avoid embolism and kept immersed (avoiding the wetting of leaves) for 24 h at 4 °C to ensure full leaf hydration [17].

### Drying experiments

Full hydrated leaves were covered with a dark plastic container and were allowed to dry slowly at room temperature. During this dehydration process, leaf mass and ultrasound measurements were repeatedly obtained in a sequential manner to achieve different levels of leaf water status. Afterwards, leaves were introduced in a stove (48 h, 80 °C) to obtain the leaf dry mass (DM). Leaf mass was measured with a precision balance (Precisa XT 220A) right before the acquisition of NC-RUS measurements. Experiments were performed on a set of 31 fully mature leaves. Around nine paired measurements of leaf mass and ultrasonic parameters were performed in each leaf. Finally, a set of 280 paired measurements were obtained.

### RWC calculation

Relative water content (RWC) was calculated following the expression: RWC = (FM − DM)/(TM − DM), where TM in the leaf turgid mass obtained at the beginning of the dehydration process, FM is the sample fresh mass measured at any moment of the process and DM is the leaf dry mass obtained as explained above.

### Pressure–volume analysis

p–v relationships were determined using a pressure chamber (Model 600 Pressure Chamber Instrument, PMS Instrument Co., Albany, OR, USA) and following the free-transpiration method described in previous studies [31–34]. The water relations parameter calculated as a mean and standard error of individual values was the RWC at the TLP.

### Non-contact resonant ultrasound spectroscopy measurements (NC-RUS)

**Fig. 4:**
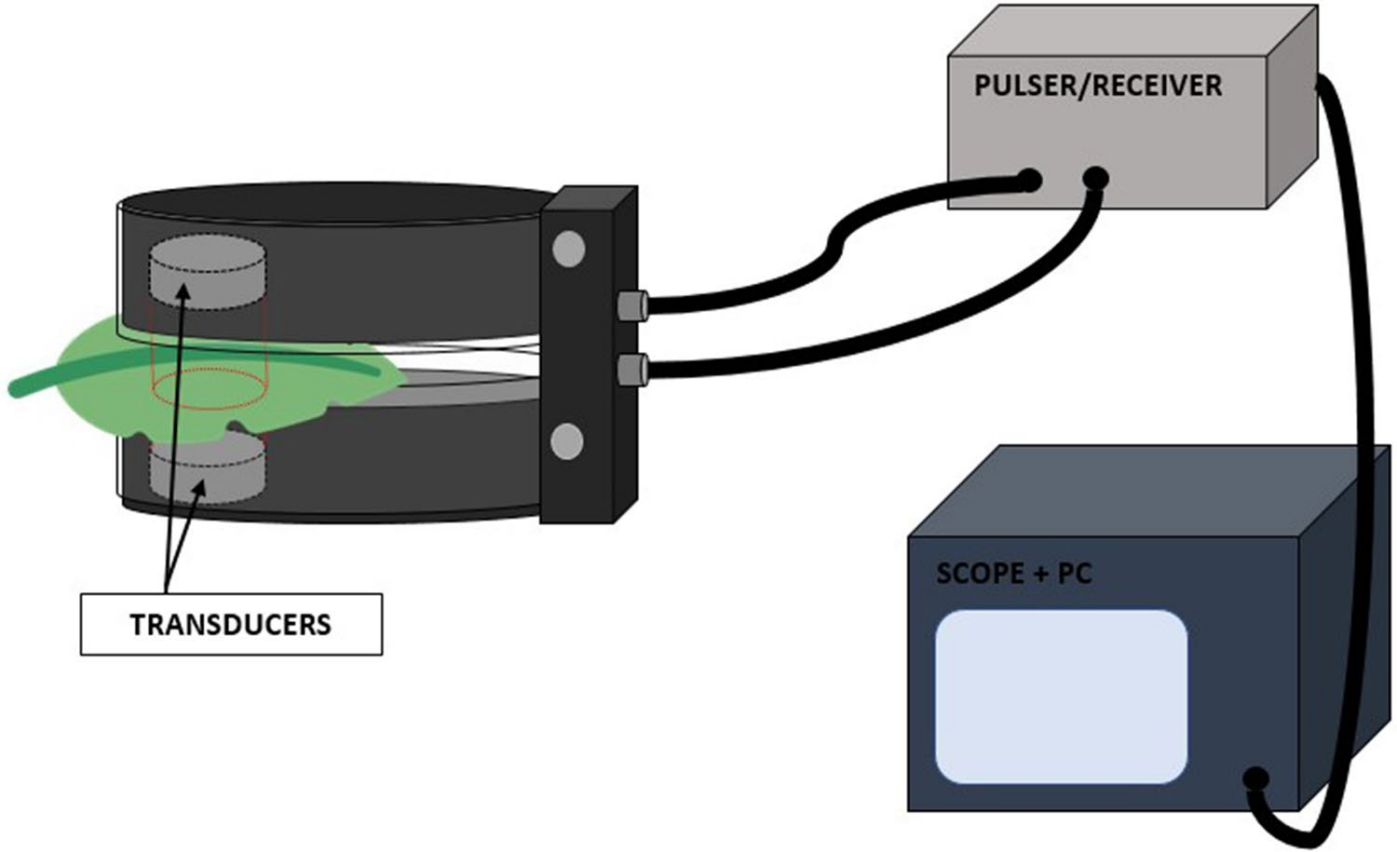
Diagram of the non-contact resonant ultrasound spectroscopy experimental setup

The NC-RUS technique is well described and in the literature [16, 35, 36] and schematically depicted in Fig. 4. In this case, the experimental setup consists of three pairs of air-coupled transducers developed, designed and built at CSIC lab. Frequency bands are 0.15–0.35, 0.35–0.95 and 0.5–1.6 MHz, peak sensitivities of − 25, − 30, − 32 dB, and active area diameters of 20, 15 and 10 mm, respectively [37, 38]. Transmitter and receiver are facing each other at a distance of 5 cm while embedded in a u-shaped holder specifically designed for these purposes. The leaf is located in a slot in between them at normal incidence. A commercial pulser/receiver (5077PR, Olympus, Houston, TX, USA) was used to drive the transmitter with a 200 V amplitude square semicycle tuned to the transducers centre frequency and to amplify and filter the electrical signal provided by the receiver transducer (up to 40 dB and low pass filtered: 10 MHz). The signal was then sent to a digital oscilloscope (TDS5054, Tektronix, Beaverton, OR, USA), the bandwidth set at 20 MHz and the acquisition in averaged mode (between 80 and 120 samples). Samples were digitized at 2, 5 and 10 MS/s, for measurements in the 250-, 650- and 1000 kHz bands, respectively, and at 8 bit (vertical). Afterwards, the signal was transferred to the oscilloscope PC, where a Matlab (The MathWorks, Inc., Natick, Massachusetts, United States) script applied Fast Fourier Transform (FFT) to it. Prior to every measurement, a calibration consisting in a measurement without any sample in between the transducers was taken. After measuring the leaf, we subtracted the calibration spectra from the measurement both in magnitude and phase in order to obtain the transmission coefficient. Once the leaf was measured using the three pairs of transducers, the magnitude and phase of the transmission coefficients were concatenated sorted by frequency.

### NC-RUS data annotation

NC-RUS transmission coefficient spectral data of leaves were annotated with their corresponding real RWC values, measured experimentally as previously explained. These annotations served as reference standard for both training and evaluation purposes.

### NC-RUS data preparation

Discrete magnitude and phase values measured by NC-RUS creating the transmission coefficient spectra were first interpolated to conform continuous transmission coefficient spectra between 100 kHz and 1.6 MHz. Seven different interpolation methods were used (linear, cubic, splines, nearest neighbors, next, previous and Akima), creating disturbed versions of collected data, therefore providing a strategy for data augmentation increasing the amount of available samples (×7). These interpolated versions served as new real samples since the small perturbations could be obtained at acquisition time. We set a common frequency reference system for all samples (100 kHz–1.6 MHz) and a sampling rate of 2.5 kHz to generate the fixed-size magnitude and phase input data (601 values each). Therefore, a total of 1960 spectral samples of size [601 × 2] were used in this work.

Additionally, we also extracted four parameters whose relevance is known from previous works. They are: the maximum value of the magnitude of the transmission coefficient, the phase and the frequency at which this maximum is located and the bandwidth of the first resonance peak (measured as the normalized separation of the frequencies with − 6 dB from the central frequency with maximum magnitude) [35].

### Machine-learning strategy

Measurements were grouped by leaf, forming 31 groups with variable amount of data representing different NC-RUS measurements at several time points (with their corresponding different RWC values) obtained from the same leaf. A leave-one-out cross-validation (CV) strategy using these groups was followed to train and evaluate a model for the prediction of RWC values from NC-RUS measurements. Therefore, in each round of the cross-validation procedure, measurements coming from 30 leaves were used for training purposes, and the evaluation was performed over all measurements from the leaf that is left. We named this strategy as leaf-one-out CV (leafOO-CV). It represents a realistic practical scenario where a RWC-prediction model would be used to estimate the RWC of a completely new leaf that was never used for training that model.

A deep learning approach using one-dimensional (1D) convolutional neural networks (CNN) was used to create a regression model able to predict RWC from magnitude and phase NC-RUS spectral data, with the objective of fully using all the NC-RUS available information, and also exploiting the non-independent relationship that exist between signals measured in nearby frequencies (Fig. 3). Additionally, a traditional machine-learning approach based on random forest (RF) [39, 40] was followed for comparison purposes, using the four NC-RUS-derived parameters to construct the prediction model.

### Convolutional neural network (CNN)

The architecture of the CNN comprised a total of 18 layers, including 3 1D-convolutional layers using (17, 55, 70) kernels with sizes (11, 7, 11), respectively, and 3 fully connected layers with (25, 25, 1) nodes, respectively, as depicted in Fig. 3a. Batch normalization and rectified linear transformation were used after each convolutional layer. Max pooling layers with window size 2 were used after the last two convolutional blocks. Dropout layers after the first 2 fully connected layers deactivate some neurons randomly with a probability of 30%. Finally, a regression layer using mean-squared-error as loss function provides the prediction of RWC value as output. The network was trained during 350 epochs with a minibatch size of 32 using stochastic gradient descent with momentum (SGDM) optimizer, 0.0875 as initial learning rate with 3 drops of factor 10, L2 regularization term of 1.25e−07, and momentum of 0.8247.

### Random forest (RF)

The more traditional RF machine-learning approach that we tested bagged an ensemble of 400 regression trees using bootstrap samples. We used sampling with replacement, half of the number of variables for each decision split, and a minimum number of 3 observations per tree leaf [39].

### Training strategy

The imbalanced learning problem, caused by the huge dominance of RWC values close to 1 in our dataset, limited the proper construction of regression models able to generalize estimations in the whole range of possible RWC measurements [41]. In order to avoid bias on the learning process and the disregarding of lower RWC values in the models, a balancing of initial training data was carried out. A density-dependent downsampling of the samples in the training set was performed by equalization of corresponding RWC values [42], applying a random subsampling that outputs a set of samples with nearly uniform distribution of their RWCs. As consequence, only some interpolated versions of NC-RUS measurements were kept for training purposes (train-set). The rest were aimed for pseudo-validation since these samples are not independent on the training set conformed by some of their interpolated siblings, and also samples corresponding to different measures but from the same leaf. This balance of training set was applied independently in each round of leafOO-CV.

### Evaluation of results

Prediction of RWC values were performed at each leafOO-CV round in samples corresponding to the leaf that was left from training, using the correspondent CNN and RF regression models. To evaluate the goodness of RWC estimations, root mean square errors (RMSE) were calculated. A global RMSE, together with the Pearson’s linear correlation coefficient (R), were reported using final RWC-predictions of all samples from the 31 testing leaves. Additionally, we also extracted RMSE and R values obtained after grouping and computing the mean of predictions for the seven interpolated versions of NC-RUS measurements (a unique value per NC-RUS measurement acquired).

## Supplementary information

**Additional file 1: Figure S1.** Statistical analyses. a) Boxplots comparing interpolation methods for prediction of RWC values with CNN (left) and RF (right) approaches. Prediction errors obtained with each approach are not statistically different when using data preprocessed with different interpolation methods (repeated measures ANOVA with both Bonferroni and Tukey-Kramer multiple comparison tests). b) Boxplot displaying RMSE values computed individually on each leaf confirm the superior performance of CNN method for estimation of RWC values (paired-sample t-test, **p-value < 0.005).

## Data Availability

The datasets analysed during the current study are available from the corresponding author on reasonable request.

## Abbreviations

NC-RUS: non-contact resonant ultrasound spectroscopy
RWC: relative water content
CNN: convolutional neural network
RF: random forest
