# Supplementary figures and images for: Instantaneous and non-destructive relative water content estimation from deep learning applied to resonant ultrasonic spectra of plant leaves
Source: Plant Methods. 2019 Nov 7;15:128. doi: 10.1186/s13007-019-0511-z (PMC6836334; doi:10.1186/s13007-019-0511-z)

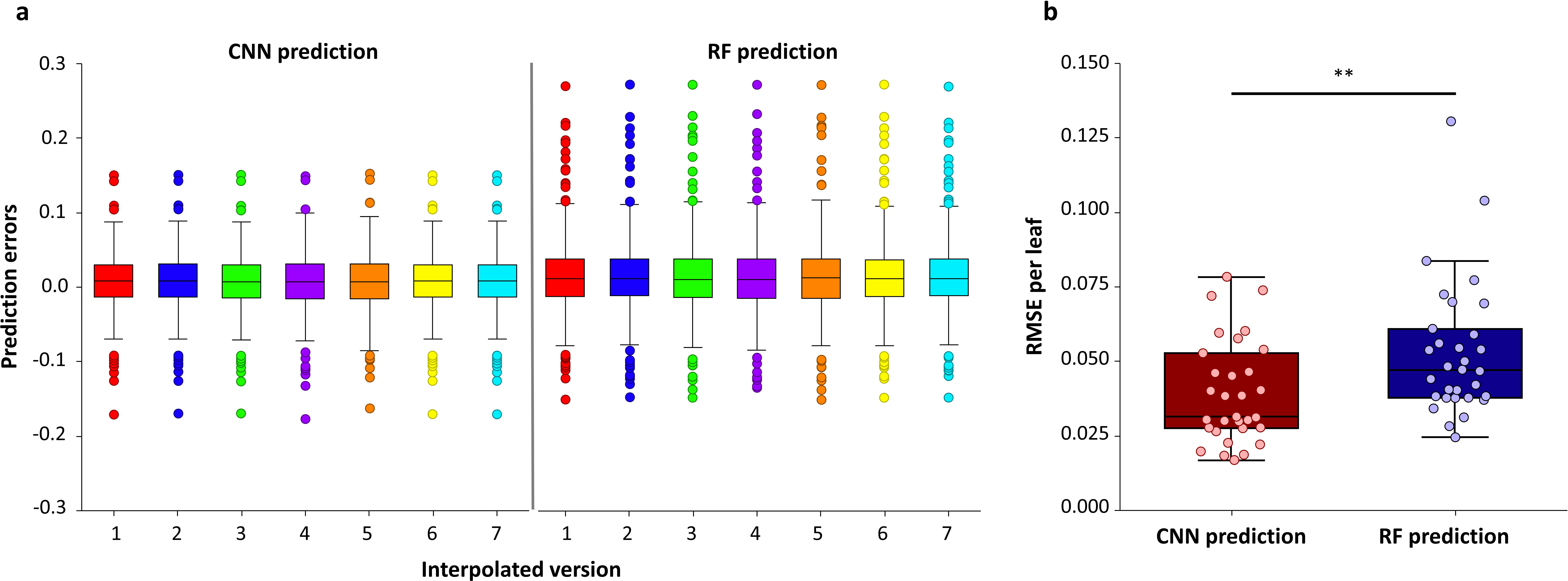

Supplement: Supplementary file 1 — Additional file 1: Figure S1. Statistical analyses. a) Boxplots comparing interpolation methods for prediction of RWC values with CNN (left) and RF (right) approaches. Prediction errors obtained with each approach are not statistically different when using data preprocessed with different interpolation methods (repeated measures ANOVA with both Bonferroni and Tukey-Kramer multiple comparison tests). b) Boxplot displaying RMSE values computed individually on each leaf confirm the superior performance of CNN method for estimation of RWC values (paired-sample t-test, **p-value < 0.005). [file 13007_2019_511_MOESM1_ESM.jpg]
